# Cardiac Natriuretic Peptide Profiles in Chronic Hypertension by Single or Sequentially Combined Renovascular and DOCA-Salt Treatments

**DOI:** 10.3389/fphys.2021.651246

**Published:** 2021-05-25

**Authors:** Carolina S. Cerrudo, Susana Cavallero, Martín Rodríguez Fermepín, Germán E. González, Martín Donato, Nicolás M. Kouyoumdzian, Ricardo J. Gelpi, Cecilia M. Hertig, Marcelo R. Choi, Belisario E. Fernández

**Affiliations:** ^1^Facultad de Farmacia y Bioquímica, Cátedras de Fisiopatología y Anatomía e Histología, Universidad de Buenos Aires, Buenos Aires, Argentina; ^2^Facultad de Medicina, CONICET, Instituto de Fisiopatología Cardiovascular, Universidad de Buenos Aires, Buenos Aires, Argentina; ^3^Instituto Alberto C. Taquini de Investigaciones en Medicina Traslacional (IATIMET), Universidad de Buenos Aires, CONICET, Buenos Aires, Argentina; ^4^Instituto de Ingeniería Genética y Biología Molecular (INGEBI), CONICET, Buenos Aires, Argentina; ^5^Instituto Universitario de Ciencias de la Salud, Fundación H. A. Barceló, Buenos Aires, Argentina

**Keywords:** natriuretic peptides system, cardiac hypertrophy, renovascular hypertension, DOCA-Salt hypertension, rat models, atrial natriuretic factor, B type natriuretic peptide

## Abstract

The involvement of natriuretic peptides was studied during the hypertrophic remodeling transition mediated by sequential exposure to chronic hemodynamic overload. We induced hypertension in rats by pressure (renovascular) or volume overload (DOCA-salt) during 6 and 12 weeks of treatment. We also studied the consecutive combination of both models in inverse sequences: RV 6 weeks/DS 6 weeks and DS 6 weeks/RV 6 weeks. All treated groups developed hypertension. Cardiac hypertrophy and left ventricular ANP gene expression were more pronounced in single DS than in single RV groups. BNP gene expression was positively correlated with left ventricular hypertrophy only in RV groups, while ANP gene expression was positively correlated with left ventricular hypertrophy only in DS groups. Combined models exhibited intermediate values between those of single groups at 6 and 12 weeks. The latter stimulus associated to the second applied overload is less effective than the former to trigger cardiac hypertrophy and to increase ANP and BNP gene expression. In addition, we suggest a correlation of ANP synthesis with volume overload and of BNP synthesis with pressure overload-induced hypertrophy after a prolonged treatment. Volume and pressure overload may be two mechanisms, among others, involved in the differential regulation of ANP and BNP gene expression in hypertrophied left ventricles. Plasma ANP levels reflect a response to plasma volume increase and volume overload, while circulating BNP levels seem to be regulated by cardiac BNP synthesis and ventricular hypertrophy.

## Introduction

Hemodynamic overload is a major determinant of the cardiac morphometric and functional response in cardiovascular diseases. Hypertension is a leading cause of congestive heart failure around the world. Elevation of the blood pressure results in left ventricular hypertrophy, an independent risk factor for cardiovascular mortality (González et al., 2018; Polak-Iwaniuk et al., 2019; Yildiz et al., 2020). Remodeling of the heart can display a spectrum of geometric patterns as a result of complex interactions between pressure and volume overload; i.e., in aortic stenosis, concentric hypertrophy due to pressure overload evolves toward a dilated eccentric pattern and heart failure (Ganau et al., 1992; Opie et al., 2006). Several animal models of hypertension triggered mainly by pressure and/or volume stimuli have been used to address the role of hemodynamic overload in cardiac hypertrophy (Fenoy et al., 1997; Dobrzynski et al., 2000; Capuano et al., 2002), reviewed in Lerman et al., 2019); however, little is known about the mechanisms involved in the transition and the reversibility from one type of overload to another.

The natriuretic peptide (NP) family includes atrial (ANP) and B-type (BNP) natriuretic peptides that are secreted by the heart and are important regulators of cardiovascular homeostasis. They act on target organs through guanylyl cyclase- coupled receptors to induce diuresis, natriuresis and vasodilation, thus reducing cardiac preload and afterload in response to stress. They also modulate cardiac growth and display anti-inflammatory and anti-fibrotic properties (Nakagawa et al., 2019; Goetze et al., 2020). Gene expression of ANP and BNP is also induced in the adult heart as part of the fetal reprogramming associated to pathological hypertrophy (Sergeeva and Christoffels, 2013). C-type natriuretic peptide was traditionally considered the endothelial NP, but it was more recently implicated in the cardiac response to heart failure (Moyes et al., 2020). Circulating levels of ANP and BNP are used as clinical biomarkers with important diagnostic and therapeutic implications in hypertension and congestive heart failure (Burnett et al., 1986; Ruskoaho, 2003; Puyó et al., 2005; Rubattu et al., 2019). Elevated circulating levels are related to increased atrial wall stretch and ventricular synthesis due to left ventricular hypertrophy (Goetze et al., 2020); however, the response of ANP and BNP throughout transitions between pressure and volume overload is not well characterized.

Our lab previously described the combination of treatments to induce pressure and volume overload in defined times and sequences, in order to resemble the natural evolution of ventricular function in hypertensive disease and to evaluate the differential behavior of ANP and BNP (Cavallero et al., 2007, 2010). We used two established experimental models: renovascular hypertension (RV), and DOCA-salt hypertension (DS). RV is characterized by normal circulating renin levels (Seto et al., 1984) and an early transient increase of the plasmatic volume (Baker et al., 1990; Edmunds et al., 1991), followed by an increase of peripheral resistance that maintains elevated blood pressure (Brody et al., 1983; Fenoy et al., 1997; Lerman et al., 2019). In contrast, DS model is predominantly characterized by volume overload (Iyer et al., 2010; Ndisang and Jadhav, 2010; Lerman et al., 2019), low renin levels (Gavras et al., 1975; Makrides et al., 1988) and high circulating catecholamine, angiotensin II and endothelin-1 levels (Bianciotti and de Bold, 2000, 2001, 2002). As a consequence of volume expansion, cardiac stretch induces cardiomyocyte hypertrophy (Ogawa et al., 1999; Basting and Lazartigues, 2017). In our previous studies, we established models of cardiac hypertrophy by sequential combination of RV followed by DS, or the inverse sequence along a 4-week period. We reported that in a short-term setting, the lately applied overload stimulus determines the remodeling and cardiomyocyte hypertrophic pattern in the sequentially combined models, and moreover, that ANP gene expression but not plasma ANP correlates with volume overload in DS model (Cavallero et al., 2007, 2010).

We hypothesized that ANP and BNP could be used as biochemical markers for volume and pressure overload, respectively, and that a prolonged overload would be required to display differential profiles of ANP and BNP, and these would help to characterize the transition from one hypertrophic pattern to the other. Therefore, the aim of the present study was to evaluate the effect of a chronic hypertensive process in rats with RV and DS procedures along 6 to 12 weeks of treatment, as well as in rats subjected to combination of RV and DS in different sequences of induction. We measured the modifications of systolic blood pressure, cardiac hypertrophy indexes, cardiac function and the synthesis and secretion of ANP and BNP.

## Materials and Methods

### Animals

Male Sprague-Dawley rats weighing 180–220 g were obtained from the Animal Care Facility of the School of Pharmacy and Biochemistry at the University of Buenos Aires. The animals were housed in an environment with controlled temperature and humidity, 12-h light/dark cycle, and access to water and food *ad libitum* (Rodents Purina Chow, Cooperacion SRL, Argentina). The protocols were approved by the Institutional Review Board at the University of Buenos Aires.

### Experimental Design

**FIGURE 1:**
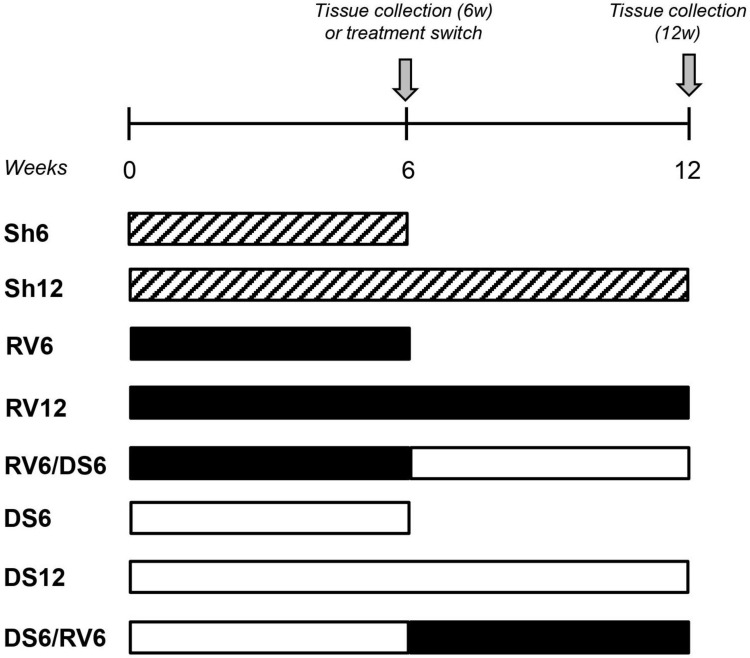
Experimental design to study single and combined hypertensive models. Diagonally striped columns: sham-operated controls, Sham 6 weeks (Sh6) and Sham 12 weeks (Sh12). Solid black columns: renovascular hypertension 6 weeks (RV6) or 12 weeks (RV12). Solid white columns: DOCA-salt hypertension 6 weeks (DS6) or 12 weeks (DS12). Combined treatments: RV6/DS6 (black/white) and the inverse sequence DS6/RV6 (white/black). The switch occurs after 6 weeks from the beginning of treatment. Appropriate sham animals were studied for each group and pooled into two groups at 6 weeks (Sh6) and 12 weeks (Sh12).

The experimental design is shown in Figure 1. Animals with renovascular (RV) or DOCA-salt (DS) hypertension were studied after 6 weeks (groups RV6 and DS6) or 12 weeks of treatment (RV12 and DS12). Groups receiving only RV or DS treatment are also referred to as “single treated groups.” Animals subjected to combined treatment consisting of 6 weeks of either RV or DS and then switched to 6 weeks of the alternate treatment (DS or RV, respectively) were studied at 12 weeks. These groups were called RV6/DS6 and DS6/RV6 or “combined treatment groups.”

Appropriate sham animals for each experimental group were included (see detailed description of procedures below). Sham animals for RV6 and DS6 groups did not show any statistical difference among them for several parameters (Supplementary Table 1) and were therefore pooled into one group, Sham 6 weeks (Sh6). Similarly, sham animals for single or combined procedures at 12 weeks did not show any statistical difference and were pooled into one group, Sham 12 weeks (Sh12). The number of animals (*n*) was 10–12 for experimental groups and 12–18 for sham groups.

### Renovascular Hypertension

The surgical procedures were performed as previously described (Puyó et al., 2005). After being anesthetized with ketamine (80 mg/kg)/xylazine (2.5 mg/kg), the left kidney was removed, and a silver clip with a 0.28 mm gap was placed on the right renal artery. For sham-operated rats, flank incisions were made to expose the kidneys and the renal artery without clipping the vessel. The animals were studied after 6 weeks (RV6) or 12 weeks (RV12). Animals in which the clip was observed outside the right renal artery at the time of sacrifice were excluded from the analysis.

### DOCA-Salt Hypertension

Rats were anesthetized as described above and subjected to left nephrectomy (Cavallero et al., 2007; Iyer et al., 2010). They received weekly injections of deoxycorticosterone acetate (DOCA, 30 mg/kg; Sigma, St. Louis, MO) suspended in sesame oil and were supplied with 1% W/V NaCl in the drinking water. Sham animals were subjected to the same surgical procedure where the kidney was exposed but not removed, and received sesame oil injections and tap water to drink. The animals were studied after 6 weeks (DS6) or 12 weeks (DS12).

### Combination of RV and DS Models

#### RV6/DS6

Six weeks after induction of RV treatment, some animals were randomly selected to undergo renal artery declipping. The clips were removed carefully by a surgery performed close to the initial incision. Upon recovery, rats received DOCA injections and 1% NaCl treatment during six additional weeks. Sham animals were subjected to simulated surgery, vehicle injections and drank tap water.

#### DS6/RV6

After 6 weeks of DOCA-salt treatment, a subgroup of rats was given no further DOCA injections or NaCl and underwent surgery for right renal artery constriction with 0.28 mm clip for six additional weeks. This group was named DS6/RV6. Sham animals discontinued vehicle injections and were subjected to simulated surgery.

### Systolic Blood Pressure Measurement

Systolic blood pressure was measured in conscious animals using the standard tail-cuff method (Blood Pressure Analysis System, Hatteras Instruments, Cary, NC, United States). All measurements were performed between 9:00 am and 1:00 pm, after 3–5 days of training.

### Plasma and Tissue Processing

At the end of the experimental period, blood samples were obtained from the abdominal cava vein under anesthesia and immediately placed into ice-chilled plastic tubes containing 15% EDTA. Then, a solution of KCl 1 mol/L was injected to induce diastolic arrest. The hearts were immediately removed, washed in cold phosphate buffered saline, blotted and weighed. Heart samples were carefully dissected by the same operator and the four cardiac chambers were weighed individually. The interventricular septum was included with the left chamber. Samples were frozen in liquid nitrogen and kept at −80°C for analysis.

The ratio between heart weight and body weight (HW/BW) was calculated to evaluate cardiac hypertrophy. The ratios for each individual chamber weight to body weight were also calculated. LVW/BW and RVW/BW indicated left and right ventricular hypertrophy, and LAW/BW and RAW/BW indicated left and right atrial hypertrophy, respectively.

### Plasma ANP and BNP Measurement

Atrial and BNP were extracted from plasma samples as previously described (Cavallero et al., 2007, 2010). Radioimmunoassay (RIA) was performed using commercially available kits to detect rat ANP and BNP-45 (Phoenix Pharmaceuticals, Burlingame, CA, United States).

### RNA Isolation and Northern Blot Analysis

Total RNA was isolated from left ventricular samples using Trizol (Invitrogen, Carlsbad, California, United States) and subjected to Northern Blot analysis as previously described (Cavallero et al., 2007). Scanning values for ANP and BNP mRNA were normalized to glyceraldehyde 3-phosphate dehydrogenase (GAPDH) mRNA.

### Transthoracic Echocardiography

Echocardiography was performed using an Acuson Sequoia C512 Ultrasound System with a 14-MHz linear transducer. Echocardiographic studies were performed under light anesthesia using ketamine (35 mg/kg) plus xylazine (5 mg/kg). The chest was shaved and the animal was positioned on a warm pad. Electrode needles were connected to each limb, and the electrocardiogram was simultaneously recorded. Rats were imaged in a shallow left lateral decubitus position. Two-dimensional parasternal short-axis imaging plane were used to obtain M-mode tracings at the level of the papillary muscles. LV internal dimensions and LV wall thickness were determined at systole and diastole. End-diastolic measurements were taken at the maximal LV diastolic dimension, and end systole was defined as the time of the most anterior systolic excursion of the posterior wall. Measurements were taken from three consecutive beats for each rat. Transmitral Doppler inflow waves were used to measure peak early diastolic filling velocity (E wave), peak filling velocity at atrial contraction (A wave), and the ratio between them (E/A) as well as isovolumetric relaxation time (ms), assessing diastolic function as previously described (Gao et al., 2011; González et al., 2016).

Systolic function was evaluated from LV dimensions by the cubed method as percentage of LV ejection fraction (LVEF): LVEF (%) = [(LVEDD3–LVESD3)/LVEDD3] × 100, where LVEDD and LVESD are LV end-diastolic diameter and LV end-systolic diameter, respectively. Diastolic and systolic wall stress was calculated by using hemodynamic and echocardiographic measurements according to Laplace’s law, where Diastolic stress = LV End-diastolic pressure x LV-diameter in diastole/2 × diastolic posterior wall thickness, and Systolic stress = LV systolic pressure × LV-diameter in systole/2 × posterior wall thickness in systole.

### Statistical Analysis

All data are expressed as mean ± standard error of the mean (SEM). Statistical analysis was performed across all eight groups by one-way ANOVA, using GraphPad Prism software (GraphPad Software Inc., San Diego, CA, United States). For multiple comparisons, we performed Tukey-Kramer post-test comparing the mean of each group with the mean of every other group to identify significant differences. *P*-values less than 0.05 were considered statistically significant and are indicated in the figures and tables.

We created the correlation analysis plots using the mean values of each experimental group for the measured variables. The linear Pearson correlation was used for correlation studies. Fisher’s *Z* test was used to compare two correlation coefficients (Marino et al., 2008). *P*-values of 0.05 or less were considered statistically significant.

## Results

### Systolic Blood Pressure

**FIGURE 2:**
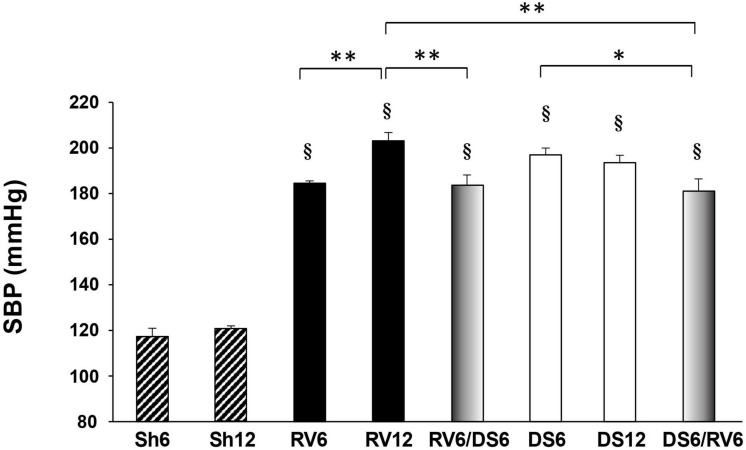
Systolic blood pressure. The groups are indicated as described in Figure 1. Diagonally striped bars: Sham controls; black solid columns: RV treatment; white solid columns: DS treatment. Combined group RV6/DS6 is shaded black to white from left to right, and the inverse sequence DS6/RV6 is shaded white to black from left to right. Results are expressed as mean ± SEM, *n* = 10–18. Differences versus vs time- matched sham group (Sh6 or Sh12) are indicated on top of the column; differences among experimental groups are indicated within brackets: *P < 0.05, **P < 0.01, ^§^*P* < 0.001.

All experimental animals regardless of the treatment became hypertensive after 6 and 12 weeks compared to sham animals (Figure 2). Single RV and DS treatments induced different temporal profile of SBP response. SBP increase in response to RV treatment was time-dependent, with higher levels at 12 weeks than at 6 weeks. In contrast, SBP increased in response to DS treatment at 6 weeks, but did not further increase after 12 weeks. We measured SBP measured after 2, 4, 6, and 12 weeks of single RV or DS treatment (Supplementary Figure 1). The time course study of SBP further evidences a continued steep increase in RV toward 12 weeks, in contrast to the plateau observed in DS from 6 to 12 weeks.

Comparing both treatments, DS6 and DS12 groups reached similar blood pressure values to RV12. The fact that only 6 weeks of DS treatment were equally effective as 12 weeks of RV suggests that DS treatment determines an earlier increase in SBP when compared with RV.

Both groups with combined RV and DS developed similar degree of hypertension regardless the sequence of treatment (mmHg: RV6/DS6: 184 ± 4; DS6/RV6: 181 ± 5; see shaded columns in Figure 2). Both combined groups presented lower SBP than single treated groups at 12 weeks (mmHg: RV12: 203 ± 4; DS12: 193 ± 4), which only reached statistical significance compared to RV12. In addition, in a subset of animals undergoing combined treatments we followed up the increase in SBP over time measured at 3, 6, 9, and 12 weeks of treatment (shown in Supplementary Figure 2). A transient reduction in SBP was observed at 9 weeks in both combined groups (3 weeks after withdrawal of the first treatment and after installation of the second treatment). The SBP continued to increase toward the 12-week end point, but it did not reach the SBP levels observed in the single treated groups RV12 and DS12. Taken together, these results suggest that a single type of stimulus persisting over time is more effective to induce hypertension that the sequential combination of two different types of hemodynamic stimuli, regardless the order of presentation.

We also verified if the withdrawal of DOCA-salt after 6 weeks of treatment is able to cause regression of hypertension and cardiac hypertrophy over the following 6-week period (Supplementary Table 1). At 12 weeks SBP was only partially normalized. However, cardiac hypertrophy reversed and was not different from the sham animals. On the other hand, it has been shown extensively in the literature that removal of the renal clip in the RV model reverses the SBP increase and the cardiovascular changes in both rats and mice (Edmunds et al., 1991; Kvist and Mulvany, 2003; Gao et al., 2005).

### Cardiac Hypertrophy and Function

We calculated hypertrophy indexes and assessed functional parameters by echocardiography (Table 1 and Supplementary Figure 3). All hypertensive groups developed cardiac hypertrophy at 6 and 12 weeks as evidenced by an increase in the heart-to-body weight ratio (HW/BW) and the LV posterior wall thickness in diastole (LVPWd). DS model showed a time-dependent increase in HW/BW ratio, with cardiac hypertrophy being more pronounced in DS12 compared to DS6 groups, while in RV we did not detect significant differences between 6 and 12 weeks. In addition, cardiac hypertrophy assessed by HW/BW was more pronounced in DS groups compared to RV groups at both time points (DS6 vs RV6 and DS12 vs RV12, *P* < 0.05 in both cases). Both combined treatments RV6/DS6 and DS6/RV6 had lower HW/BW compared to the single groups RV12 and DS12, respectively.

**TABLE 1:** Hypertrophic indexes and cardiac function assessed by echocardiography.

**Group**	**HW/BW mg/g**	**LVW/BW mg/g**	**RVW/BW mg/g**	**LAW/BW mg/g**	**RAW/BW mg/g**	**LVEDD mm**	**LVPWd mm**	**Ejection fraction, %**	**Shortening fraction, %**
Sh6	2.56 ± 0.05	1.95 ± 0.04	0.43 ± 0.01	0.12 ± 0.01	0.11 ± 0.01	6.60 ± 0.11	1.43 ± 0.05	72.70 ± 0.81	36.53 ± 4.40
Sh12	2.46 ± 0.06	1.70 ± 0.04	0.52 ± 0.01	0.11 ± 0.01	0.10 ± 0.01	6.63 ± 0.32	1.47 ± 0.03	68.03 ± 1.23	33.37 ± 0.89
RV6	3.30 ± 0.11^†^	2.48 ± 0.10*	0.59 ± 0.05*	0.17 ± 0.01*	0.12 ± 0.01	6.10 ± 0.20	1.71 ± 0.05*	77.60 ± 2.21	41.49 ± 2.03
RV12	3.61 ± 0.16^‡^	2.49 ± 0.20^‡^	0.65 ± 0.04*	0.17 ± 0.01^‡^	0.13 ± 0.01*	6.45 ± 0.25	1.85 ± 0.05*	77.01 ± 2.50	40.65 ± 2.35
RV6/DS6	3.19 ± 0.05^‡^	2.36 ± 0.06^‡^	0.59 ± 0.01	0.16 ± 0.01^†^	0.10 ± 0.01^§^	6.15 ± 0.15	1.77 ± 0.03*	76.40 ± 2.40	40.01 ± 2.10
DS6	3.89 ± 0.11^‡$^	2.54 ± 0.13^‡^	0.59 ± 0.04*	0.17 ± 0.01*	0.14 ± 0.01*	6.47 ± 0.09	1.77 ± 0.07*	80.20 ± 3.10	43.83 ± 2.92
DS12	4.31 ± 0.16^‡a§^	2.58 ± 0.26^‡^	0.73 ± 0.05^†^	0.21 ± 0.01^‡a^	0.17 ± 0.01^‡a§^	7.00 ± 0.26	1.88 ± 0.07*	84.16 ± 2.90^†^	48.62 ± 3.26*
DS6/RV6	3.37 ± 0.08^‡^^c^	2.13 ± 0.11*	0.66 ± 0.03*	0.16 ± 0.01^†b^	0.12 ± 0.01*^c^	6.93 ± 0.21	1.90 ± 0.16*	71.83 ± 3.44	36.58 ± 2.69
*n*	*10–18*	*5–9*	*5–9*	*10–18*	*10–18*	*3–8*	*3–8*	*3–8*	*3–8*

Ratios are calculated in mg tissue/g body weight. LVEDD and LVPWd are calculated in mm. Values are expressed as mean ± SEM. BW, body weight; HW, heart weight; LVW, left ventricle weight; RVW, right ventricle weight; LAW, left atria weight; RAW, right atria weight; LVEDD, LV end diastolic diameter; LVPWd, LV posterior wall thickness in diastole. The number of animals per group (*n*) is indicated for each parameter. The groups are nominated as described in Figure 1. *P < 0.05, ^†^*P* < 0.01 and ^‡^*P* < 0.001 versus time-matched sham (Sh6 or Sh12), ^$^*P* < 0.01 versus RV6; ^§^*P* < 0.05 versus RV12; ^*a*^*P* < 0.05 versus DS6; ^*b*^*P* < 0.05, and ^*c*^*P* < 0.001 versus DS12.

At the individual chamber level, RV as well as DS groups developed left ventricular hypertrophy when compared to sham animals, reaching similar LVW/BW ratios at 6 and 12-weeks of treatment. Single treated hypertensive groups developed right ventricular hypertrophy (RVW/BW) at 6 and 12 weeks. All hypertensive groups also developed left atrial hypertrophy, but only DS groups showed a time-dependent increase in LAW/BW (DS12 vs DS6, *P* < 0.05). Finally, right atrial hypertrophy (RAW/BW) was observed in DS groups at 6 weeks and was even more pronounced after 12 weeks. In contrast, right atrial hypertrophy developed in RV only after 12 weeks of treatment. Taken together, these results suggest that pronounced cardiac hypertrophy elicited by DS treatment may be sustained by an earlier development of hypertrophy in the right atria. Finally, both combined treatments RV6/DS6 and DS6/RV6 had less increase in RAW/BW compared to the single groups RV12 and DS12, respectively.

Functional data obtained by echocardiography are shown in Table 1 and Supplementary Figure 3. All groups exhibited cardiovascular remodeling, with increased left ventricular wall thickness without dilation of the left ventricular chamber. The results of ejection fraction and shortening fraction show that left ventricular systolic function was preserved and none of the groups presented severe ventricular dysfunction. All hypertensive groups have compromised diastolic function as reflected by an increased isovolumic relaxation time (IVRT). At 6 weeks, DS group showed increased IVRT compared to RV6, however, at 12 weeks the IVRT did not differ between RV12 and DS12. Systolic parietal stress was elevated in DS at 6 weeks and more at 12 weeks, while in RV was increased only at 12 weeks. Furthermore, DS6/RV6 showed decreased systolic parietal stress compared to DS12. Taken together, these results indicate that DS treatment leads to an earlier compromise in both diastolic and systolic function compared to RV.

### Plasma ANP and BNP Levels

**FIGURE 3:**
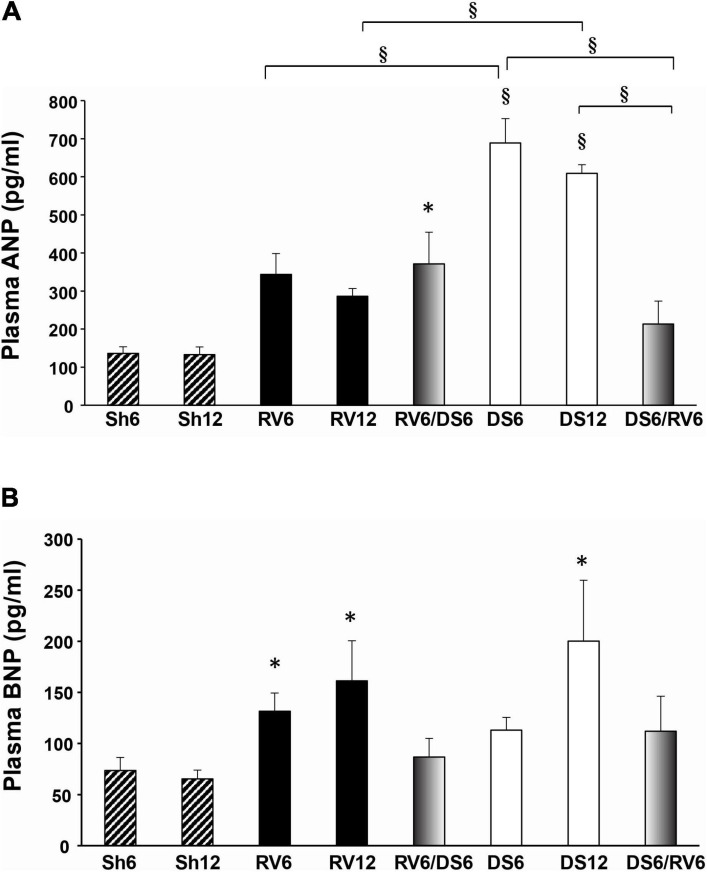
Circulating natriuretic peptide levels. Plasma ANP **(A)** and BNP **(B)**. Results are expressed as mean ± SEM. The groups are nominated as described in Figure 1, and the columns are color coded as in Figure 2. Differences versus vs time- matched sham group (Sh6 or Sh12) are indicated on top of the column (no brackets); differences among experimental groups are indicated within brackets: ^∗^*P* < 0.05, ^§^*P* < 0.001.

We measured circulating levels of ANP and BNP as indicators of cardiac hormone secretion. Plasma ANP (Figure 3A) tended to increase in RV6 and RV12 compared to sham animals, although it did not reach statistical significance. In contrast, plasma ANP levels remarkably increased in both DS6 and DS12 compared to sham, and were statistically different from RV6 and RV12, respectively.

The RV6/DS6 combined model showed ANP concentrations that were not distinguishable from RV6 or RV12; however, the inverse sequence (DS6/RV6) showed significantly blunted ANP levels compared to DS6 and DS12.

Plasma BNP levels increased in both RV6 and RV12 groups (Figure 3B), and only in DS-treated animals after 12 weeks (DS12). Both combined models RV6/DS6 and DS6/RV6 had circulating BNP levels within the normal range when measured at 12 weeks.

### Cardiac ANP and BNP Gene Expression

**FIGURE 4:**
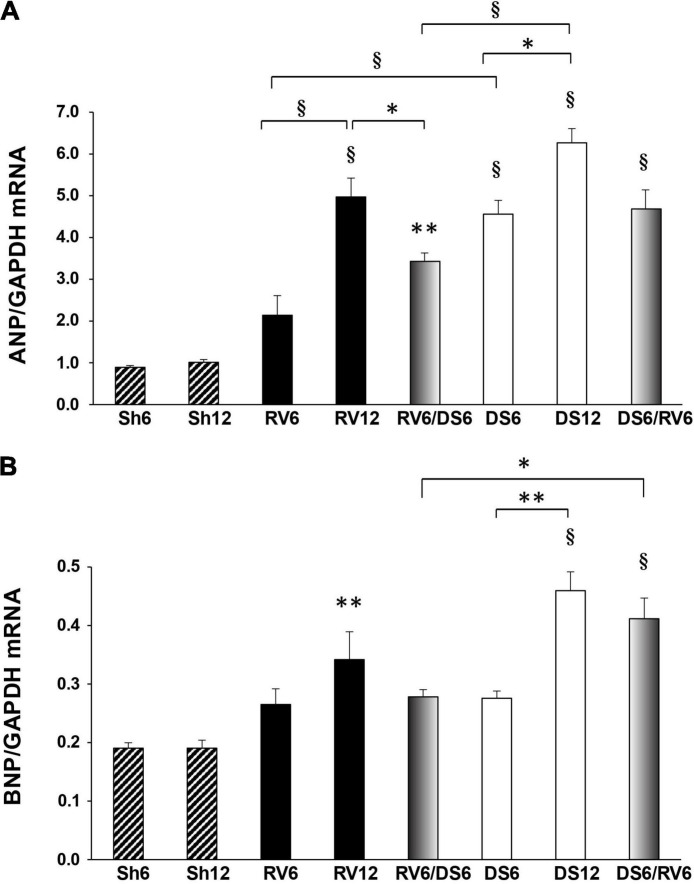
Left ventricular gene expression of ANP and BNP. ANP mRNA **(A)** and BNP mRNA **(B)** in the left ventricle. Results are expressed as mean ± SEM, *n* = 3–5. The groups are nominated as described in Figure 1, and the columns are color coded as in Figure 2. Differences versus vs time- matched sham group (Sh6 or Sh12) are indicated on top of the column (no brackets); differences among experimental groups are indicated within brackets: ^∗^*P* < 0.05, ^∗∗^*P* < 0.01, ^§^*P* < 0.001. GAPDH, glyceraldehyde-3-phosphate dehydrogenase.

We evaluated ANP and BNP gene expression as an indicator of natriuretic peptide synthesis in cardiac tissues. Northern Blot analysis showed a time-dependent increase of ANP mRNA expression in the left ventricle for RV and DS, with higher values at 12 weeks in both treatments (Figure 4A). ANP gene expression was exacerbated in response to DS at 6 weeks compared to RV (4.5-fold-increase for DS6 versus 2-fold for RV6, *P* < 0.001), but only moderately at 12 weeks (6-fold-increase for DS12 versus 5-fold for RV12; the difference was not statistically different when comparing DS12 with RV12). Both combined treatments showed increased ANP mRNA levels compared to sham; RV6/DS6 showed significantly lower expression than RV12 or DS12.

Meanwhile, BNP mRNA did not significantly increase in RV6 and DS6, but increased 0.75-fold in RV12 and 1.35-fold in DS12 (Figure 4B). Only DS6/RV6 combined group had increased BNP mRNA compared to the opposite sequence RV6/DS6 and similar to DS12.

### Correlation Analysis

#### Hypertension Versus Cardiac Hypertrophy

**FIGURE 5:**
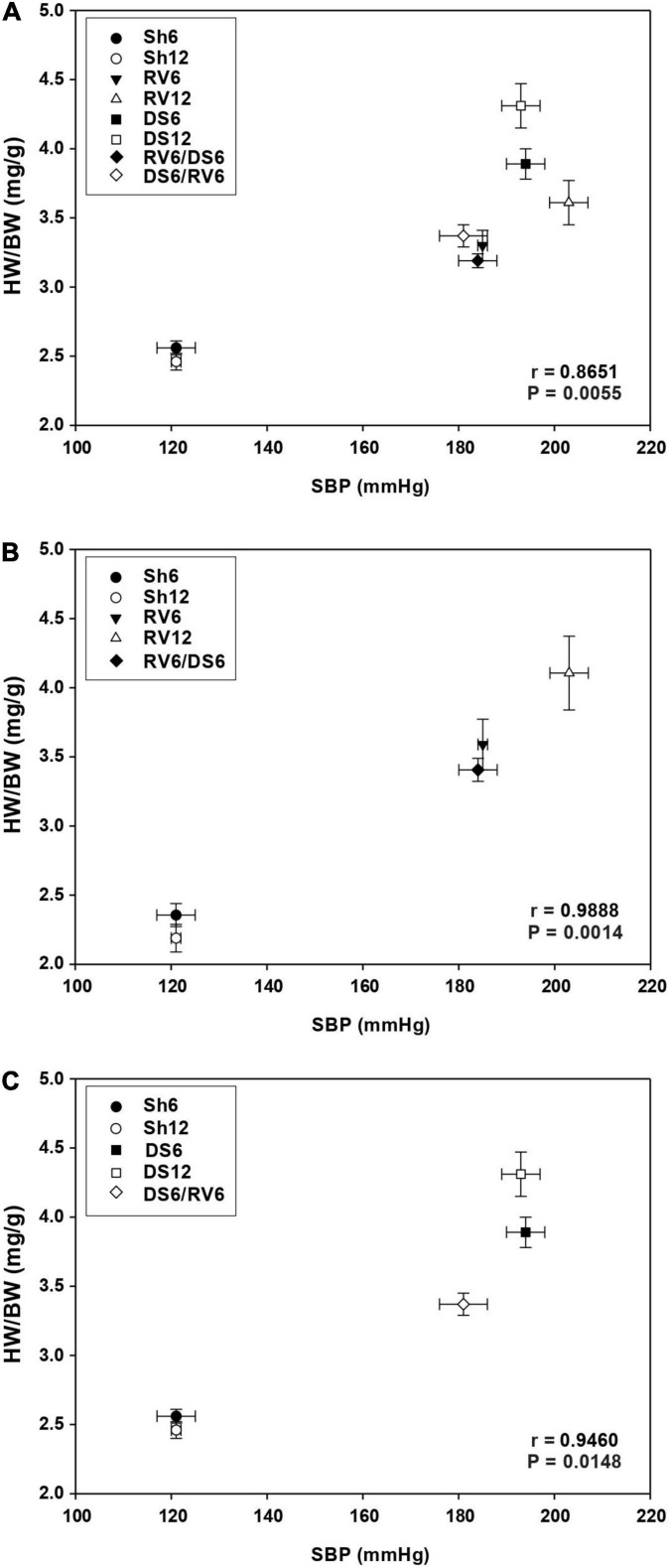
Relationship between the evolution of cardiac hypertrophy (HW/BW) and systolic blood pressure (SBP) levels. Panel **(A)** analysis including all RV and DS groups; panel **(B)** analysis including only RV groups; panel **(C)** analysis including only DS groups. Each point on the graph represents the mean ± SE for each group. The groups are nominated as described in Figure 1.

We analyzed the relationship between hypertension (SBP value) and cardiac hypertrophy (HW/BW ratio). There was a positive correlation between SBP and HW/BW when all the groups were taken together (Figure 5A) and also when the RV (Figure 5B) and DS (Figure 5C) groups are analyzed separately. The correlation was more significant in RV than in DS (Fisher’s *z* test between correlation coefficients for RV vs. DS: −4.7411, *P:* 0.0002). This result can be explained, at least in part, by the time dependent increase in SBP in response to RV treatment between 6 and 12 weeks, which is not observed in DS treatment (see Figure 2 and Supplementary Figure 1).

#### ANP and BNP Circulating Levels Versus Ventricular Gene Expression

We analyzed the relationship between plasma levels of ANP and BNP peptides and their ventricular gene expression (graphs not shown). We did not find any correlation between circulating ANP and ANP gene expression neither in DS (*r:* 0.7717, *P:* 0.1263) nor RV groups (*r:* 0.6718, *P:* 0.2143). In contrast, the concentration of circulating BNP correlated with BNP gene expression in RV groups (*r:* 0.8816; *P*: 0.0480), but not in DS groups (*r:* 0.8750; *P:* 0.0525).

#### Cardiac Hypertrophy Versus Natriuretic Peptide Ventricular Expression

**FIGURE 6:**
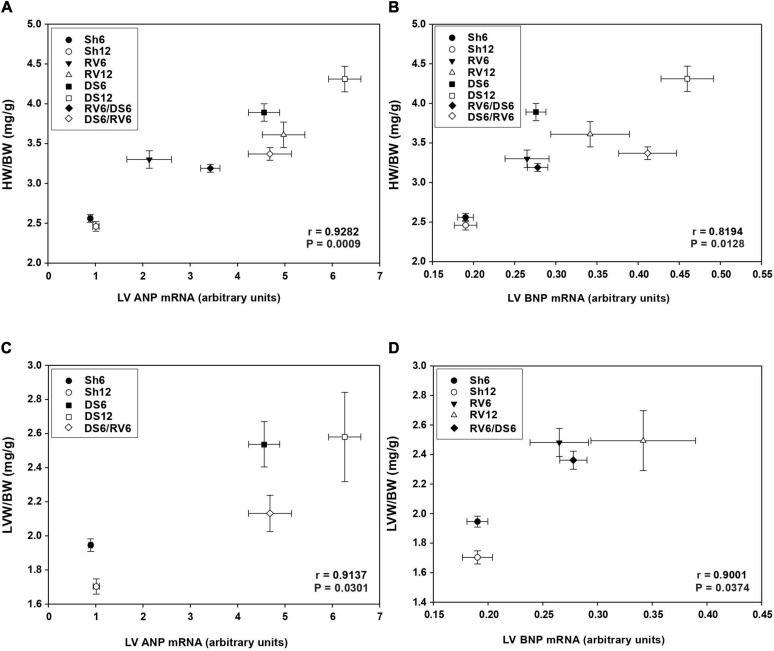
Relationship between cardiac hypertrophy (HW/BW) and left ventricular NPs mRNA expression. Panels **(A,B)** analysis for ANP and BNP mRNA, respectively, including all RV and DS groups. Panel **(C)** analysis for ANP mRNA including only DS groups; panel **(D)** analysis for BNP mRNA including only RV groups. Each point on the graph represents mean ± SEM for each group. The groups are as nominated as described in Figure 1.

Figures 6A,B show the relationship for all experimental groups between left ventricular ANP and BNP gene expression level and cardiac hypertrophy, respectively. ANP as well as BNP mRNA were positively correlated to cardiac hypertrophy expressed as HW/BW ratio.

In addition, ANP mRNA showed positive correlation with the degree of left ventricular hypertrophy (LVW/BW ratio) when all groups were analyzed together (*r*: 0.7415, *P*: 0.0355; graph not shown). Moreover, a stronger positive correlation was exhibited when the DS groups were evaluated separately (Figure 6C; Fisher’s *z* test between correlation coefficients for RV vs. DS: −2.2126, *P*: 0.0135). For the RV groups evaluated separately, the correlation was not statistically significant (*r:* 0.6353, *P*: 0.1750, graph not shown).

Finally, left ventricular BNP mRNA expression showed positive correlation with left ventricular hypertrophy only in the RV groups (Figure 6D), but not in DS groups (*r:* 0.6880, *P*: 0.1991; graph not shown).

## Discussion

Remodeling of the heart in response to persistent hemodynamic overload can lead to a spectrum of geometric, functional and hormonal patterns. We aimed to identify differential profiles of cardiac natriuretic hormones ANP and BNP in rats undergoing sequential combination of 6 weeks of renovascular followed by 6 weeks of DOCA-salt treatment or vice versa, and compared to the single treatments applied for 6 or 12 weeks. Our results show that in the models combining 6 weeks of each treatment, regardless of the sequence of presentation the overload stimulus associated to the second treatment is less effective than the first stimulus to sustain cardiac hypertrophy and to increase ANP and BNP expression. Moreover, in a chronic setting ANP correlates with volume overload while BNP correlates with pressure overload- induced cardiac hypertrophy. Thus, the combination of overload stimuli in different sequences provide an experimental tool to gain understanding about the events involved in the transition of hemodynamic states that occur along the evolution of cardiac disease.

### Systolic Blood Pressure and Cardiac Hypertrophy

The development of hypertension in response to RV was gradual and time-dependent, in agreement with previous studies in this model (Gao et al., 2005) while in DS the blood pressure reached a plateau at 6 weeks, with higher levels than RV, and persisted elevated with no further increase up to 12 weeks. Consistently, a study by Abrams et al. (2010) described distinct phases in the development of DOCA-salt hypertension: an initiation phase of rapid increase in blood pressure, followed by a development phase with a slower rise and a maintenance phase.

Renovascular model developed cardiac hypertrophy at 6 and 12 weeks, consistent with previous studies (de Simone et al., 1992; Sharifi et al., 2004; Gao et al., 2005). In DS treatment the hypertrophic process was even more pronounced than in RV, despite the lack of difference in SBP levels between 6 and 12 weeks. These results suggest that mechanisms other than SBP contribute to cardiac hypertrophy in DS. In this regard, Yokota et al. (1995) suggested that hypertension is not the initial cause for cardiac hypertrophy in DS model, given that 1 week of DS treatment was able to trigger hypertrophy even before the development of hypertension. The analysis of individual cardiac chamber hypertrophy indexes showed that right and left atrial hypertrophy in DS12 rats contributed to the difference observed in whole heart hypertrophy compared with RV.

All groups exhibited cardiovascular remodeling, with increased left ventricular wall thickness without dilation of the left ventricular chamber. The results of ejection fraction and shortening fraction show that left ventricular systolic function was preserved and none of the groups presented severe ventricular dysfunction. In addition, DS treatment leads to an earlier compromise in both diastolic and systolic function compared to RV. However, RV and DS did not show distinctive features of defined concentric or eccentric hypertrophy. Hypertensive disease per definition involves both pressure and volume overload components, therefore RV and DS are not 100% pure models for pressure and volume, respectively. Both models rather have a predominant component of pressure or volume, which leads to geometric patterns that are not completely concentric or eccentric, as it would happen in hypertrophic versus dilated cardiomyopathies of genetic origin.

Both combined models (RV6/DS6 and DS6/RV6) had lower SBP levels at 12 weeks than the single overloaded models RV12 and DS12, respectively. Cardiac hypertrophy, as well as the hypertrophy of the four chambers at 12 weeks was less pronounced in the combined treated groups than in the single treated models. A similar behavior was observed in our previous study, where we evaluated combined models at 4 weeks (Cavallero et al., 2007). These results confirm that suppression of one type of overload and substitution by another type is less effective than a single overload maintained throughout time, and that switch between treatments results in a slowdown of the hypertensive and hypertrophic process.

### Circulating ANP and BNP

The circulating ANP and BNP levels are the result of a balance between gene regulation, peptide release and clearance by NPR-C receptors and the metallopeptidase neprilysin (Rubattu et al., 2020). In addition, the levels of biologically active NPs are also regulated by cardiac and renal transmembrane proteases (corin and furin) that cleave the inactive precursors proANP and proBNP into active smaller fragments (Goetze et al., 2020). Plasma ANP and BNP levels were measured as an index of NPs cardiac secretion. Plasma ANP was increased in both RV and DS treatments after 6 and 12 weeks. In our previous studies (Cavallero et al., 2007, 2010) we reported that both treatments induced a modest increase of similar magnitude in plasma ANP of 2 and 4 weeks of treatment, and that only the ventricular gene expression of ANP, but not the plasma ANP, was indicative of volume overload at 4 weeks. In this study, plasma ANP is much more increased in response to DS than RV treatment in the chronic setting at both 6 and 12 weeks. Interestingly, when volume overload is suppressed and replaced by RV treatment (group DS6/RV6) the increased ANP levels were reverted to the level of the sham animals. Therefore, plasma ANP could be considered a marker of volume overload in the DS model in a chronic setting.

The main source of ANP in plasma and the main stimulus for the release of ANP in DS is volume overload that leads to stretching of the right atrium. In contrast, the RV model has predominantly pressure overload that mainly involves stretching of the left ventricular myocardium. These facts may explain why plasma ANP increases much more in response to DS than to RV treatment. Furthermore, these results are also in agreement with Yokota et al. who have reported a marked increase in cardiac ANP production and its secretion into the circulation in DS model (Yokota et al., 1995).

However, in the RV model when pressure overload is suppressed and replaced by DS treatment (group RV6/DS6) the ANP levels did not further increase, and showed similar levels to RV6 and RV12. A possible explanation to this observation is that after the interruption of RV model there is a partial regression of the hypertensive process (Supplementary Figure 2). Moreover, DS model is based on pharmacological treatment with DOCA injections and salt intake, which in a heart that is already hypertrophied may not generate such a pronounced response as it would happen in a normal heart. We speculate that there is a longer latency period for the RV/DS transition than for the opposite sequence DS/RV (in which the effect of the constrictor clip takes place immediately after the surgery). Further investigations will be needed to provide a definitive explanation to this lack of ANP response in the RV/DS model.

We previously showed that plasma BNP levels increased only after 4 weeks of RV treatment (Cavallero et al., 2007) and in the present study we observed elevated BNP in RV at both 6 and 12 weeks. In contrast, plasma BNP did not increase at 6 weeks and only increased after 12 weeks of DS treatment. These results suggest that BNP responds to volume overload only after a sustained exposure, and therefore we conclude that BNP plasma level is an earlier indicator for pressure overload in RV.

### ANP and BNP Gene Expression

We evaluated ANP and BNP gene expression as a more reliable index of natriuretic peptide synthesis than the tissue protein level, since the amount of tissue protein may be influenced by several processes like synthesis, storage, intragranular cleavage, clearance, and intracrine/autocrine release or endocrine secretion (Yokota et al., 1995). More recently, epigenetic mechanisms have been shown to modulate several components of the NP system (Rubattu et al., 2020).

The results obtained in the present study allow us to suggest that hypertrophic growth and NPs gene induction are processes that develop in parallel in the single-overloaded models. Our results show that DS treatment elicits an earlier induction of ANP gene expression compared to RV, since ANP mRNA was increased at 6 weeks in DS treated animals but not in RV. ANP mRNA expression markedly increased in RV and DS treatment from 6 to 12 weeks, and at 12 weeks there was no difference between RV and DS treatment. In contrast, BNP mRNA did not increase at 6 weeks in RV and DS but increased in both treatments at 12 weeks. These data are in agreement with previous reports (Yokota et al., 1990; Liu and Yoshimi, 1995; Bianciotti and de Bold, 2000, 2001; Wolf et al., 2001) and our previous studies (Cavallero et al., 2007, 2010). Moreover, mRNA expression of ANP and BNP positively correlate with ventricular weight (Kohno et al., 1992).

In RV model, the increase in gene expression was not sufficient to increase plasma levels on ANP at 12 weeks. A recent study also showed that the accumulation and release of ANP from myocyte granules depends on the pathogenesis of hypertension, and described a higher number of ANP secretory granules in a salt loading model compared to two-kidney 1-clip hypertension, providing a possible explanation for the lack of increase in plasma ANP in our RV model (Bugrova and Galkina, 2020). Another possible explanation is a differential regulation of the tissue cleavage enzymes and clearance receptors, which we have not addressed in this study.

In the combined models, left ventricular ANP mRNA expression showed intermediate levels between those observed in both single treated RV12 and DS12 and their corresponding sham groups, supporting the concept that combined models display an intermediate behavior between those observed in single models.

### Hormonal and Hypertrophic Profiles

BNP gene expression in the left ventricle was more positively correlated with left ventricular hypertrophy index only in RV groups, while ANP gene expression was positively correlated with left ventricular hypertrophy index only in DS groups. These results strongly suggest that throughout the evolution of left ventricular hypertrophy, ventricular re-expression of ANP is mainly induced in volume-overloaded DS groups and BNP in pressure-overloaded RV model. Both combined groups had a similar increase of left ventricular hypertrophy. However, left ventricular BNP expression was higher in DS6/RV6. Then, the induction of both ventricular hypertrophy and the natriuretic peptide expression in the combined treated groups would be two independent processes.

Plasma ANP did not correlate with ANP gene expression in left ventricle neither in DS nor RV groups, while plasma BNP correlated with left ventricular BNP mRNA in RV groups, but not in DS groups. These results suggest that plasma ANP levels not only are influenced by cardiac ANP secretion, but also by other tissue sources. On the other hand, circulating BNP levels in RV model seems to be regulated by cardiac BNP synthesis, which depends, among other factors, of ventricular hypertrophy. In agreement, Yokota et al. (1995) reported that while an increase in circulating levels of both ANP and BNP reflects ventricular hypertrophy, increases in plasma ANP without a concomitant increase in plasma BNP indicates atrial hemodynamic overload independently of ventricular hypertrophy.

### Combined Models

The switch RV-to-DS treatment as well as the opposite sequence DS-to-RV in chronic combined models of hypertension slowed down the progression of hypertension, the cardiac hypertrophy, and the increase of ANP and BNP expression, although the cardiac overload continued for 12 weeks. This behavior differs from the acute combined models, where we reported that the second stimulus of mechanical overload determines the evolution of hypertrophy and the synthesis and secretion of NPs (Cavallero et al., 2007, 2010). In the acute setting, hypertension and cardiac hypertrophy elicited in response to the first treatment are rapidly reverted when the treatment is withdrawn- and eventually would completely regress after 2 weeks without treatment-, thus allowing the second stimulus to rapidly predominate over the first one, and as a result the heart displays the characteristics of the second mechanical overload. However, in a chronic setting where the hypertrophic process is well established after 6 weeks of the first type of overload, the effects of the second overload may not be independent of the effects of the previous overload. As a consequence, the “new” type of hypertrophic process that takes place after the switch is slower and the heart maintains a pattern of response more similar to the first than to the last stimulus. Further experimentation will be needed in order to study the biological and molecular sequence of events developed in response to the switch pressure-volume and volume-pressure in the combined overloaded hearts.

### Study Limitations

As mentioned above, RV and DS are not pure models and have a predominant component of pressure or volume overload, respectively. We chose these models because alternative surgical procedures for pure hemodynamic overload in rats such as aortic constriction (pressure) and aortocaval shunt (volume) involve complex surgical interventions that are difficult to revert; therefore they are not amenable to perform regression and combination studies (Wu et al., 2015). In addition, in the present study we evaluated the effect of the treatments only at the end points (6 and 12 weeks). The goal of future studies will be to investigate the complex events that happen during the critical process of transitioning between models, including the behavior of the RAAS system, and this will carry critical implications to understand the progression of disease in humans.

## Data Availability Statement

The raw data supporting the conclusions of this article will be made available by the authors, without undue reservation.

## Ethics Statement

The animal study was reviewed and approved by the Institutional Review Board at the University of Buenos Aires.

## Author Contributions

CC, SC, and BF conceived and designed the study. CC, SC, and MRF performed the experiments and statistical analysis. MD, GG, and RG performed echocardiography analysis and wrote sections of the manuscript. CC and CH performed Northern Blot analysis. CC, SC, MRF, GG, NK, MC, and BF wrote the manuscript. All authors contributed to manuscript revision, read, and approved the submitted version.

## Conflict of Interest

The authors declare that the research was conducted in the absence of any commercial or financial relationships that could be construed as a potential conflict of interest.

## Supplementary Material

Supplementary Figure 1 Systolic blood pressure (SBP) in RV (upper panel) and DOCA-salt hypertension (lower panel) single treatments compared to their sham at 2, 4, 6, and 12 weeks of treatment. Results are expressed as mean ± SEM. ^∗^*P* < 0.01 and ^∗∗∗^*P* < 0.001 versus corresponding sham; ^†^*P* < 0.05 and ^‡^*P* < 0.001 versus RV2; ^§^*P* < 0.01 versus RV6: ^§^*P* < 0.05 vs. DS2; ^#^*P* < 0.05 vs. DS4.

Supplementary Figure 2 Systolic blood pressure (SBP) in single and combined RV and DS treatments measured at 3, 6, 9, and 12 weeks of treatment. Results are expressed as mean ± SEM.

Supplementary Figure 3 Ventricular function parameters. Differences versus vs time- matched sham group (Sh6 or Sh12) are indicated on top of the column; differences between experimental groups are indicated within brackets: ^∗^*P* < 0.05, ^∗∗^*P* < 0.01, and ^§^*P* < 0.001.

Supplementary Table 1 Systolic blood pressure (SBP) and hypertrophic indexes in sham animals for different groups. Ratios are calculated in mg tissue/g body weight. Values are expressed as mean ± SEM. BW, body weight; HW, heart weight; LVW, left ventricle weight; RVW, right ventricle weight; LAW, left atria weight; RAW, right atria weight. The number of animals (n) per group is indicated for each parameter. Sh-RV6 and Sh-RV12 are the sham animals for renovascular groups; Sh-DS6 and Sh-DS12 are the sham animals for DOCA-salt groups; Sh-RV6/DS6 and Sh-DS6/RV6 are the sham animals for combined groups. Animals were subjected to simulated surgeries and vehicle injections at the same time points as the experimental groups as indicated in the Methods section.

Supplementary Table 2 Regression of hypertension and cardiac hypertrophy at 12 weeks after withdrawal of DOCA-salt treatment for 6 weeks. A group of animals were subjected to left nephrectomy and DOCA/salt administration as described in the Methods section; after 6 weeks the treatment was discontinued and the parameters were evaluated at 12 weeks. Values are expressed as mean ± SEM. Number of animals (*n*) is indicated between brackets for each group. ^§^*P* < 0.001 vs corresponding sham; ^@^*P* < 0.001 vs DS6; ^$^*P* < 0.01 and ^#^*P* < 0.001 vs DS12; ^∗^*P* < 0.05 vs DS6/RV6.
