# Supplementary figures and images for: Cardiac Natriuretic Peptide Profiles in Chronic Hypertension by Single or Sequentially Combined Renovascular and DOCA-Salt Treatments
Source: Front Physiol. 2021 May 25;12:651246. doi: 10.3389/fphys.2021.651246 (PMC8185994; doi:10.3389/fphys.2021.651246)

# Supplementary Figure 1

**A**

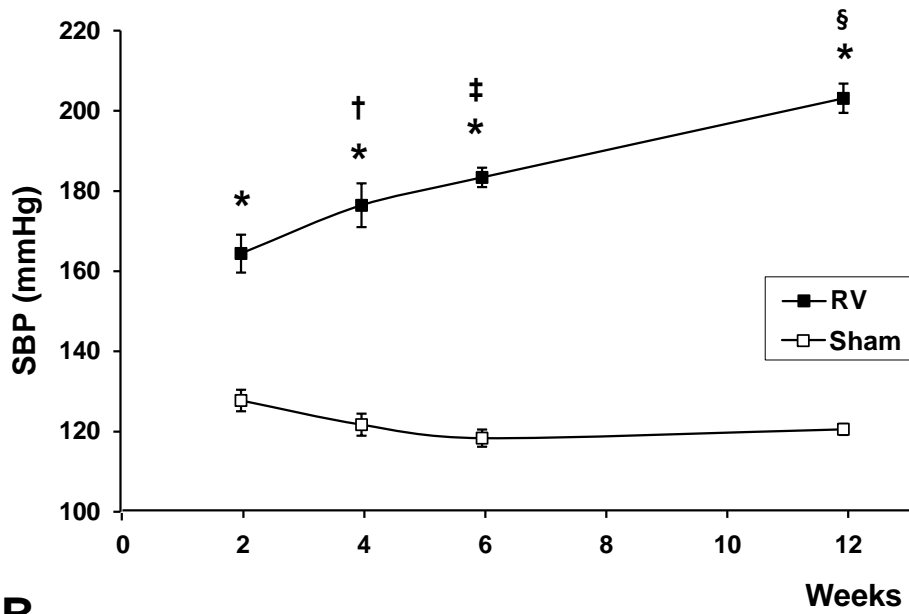

**B**

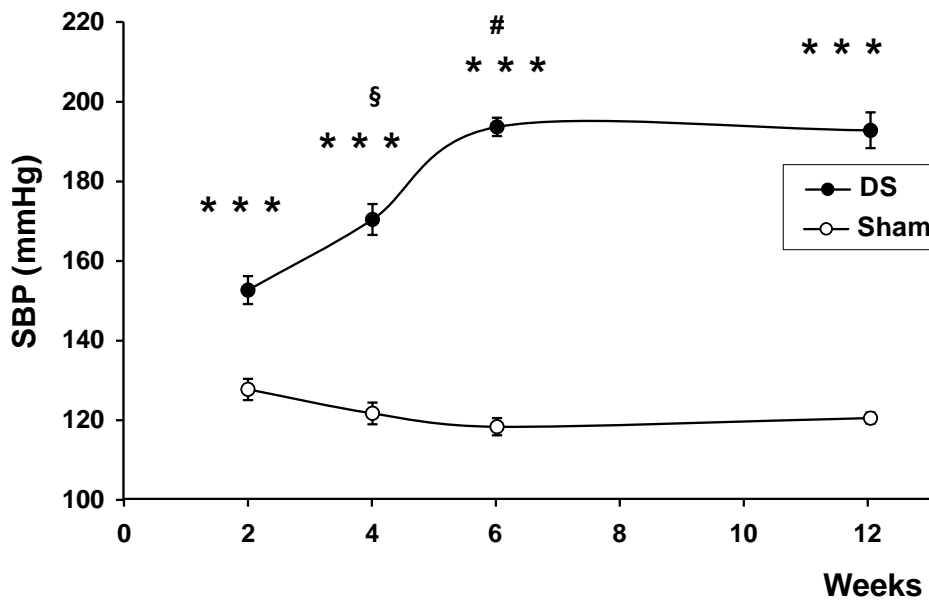

## Supplementary Figure 2

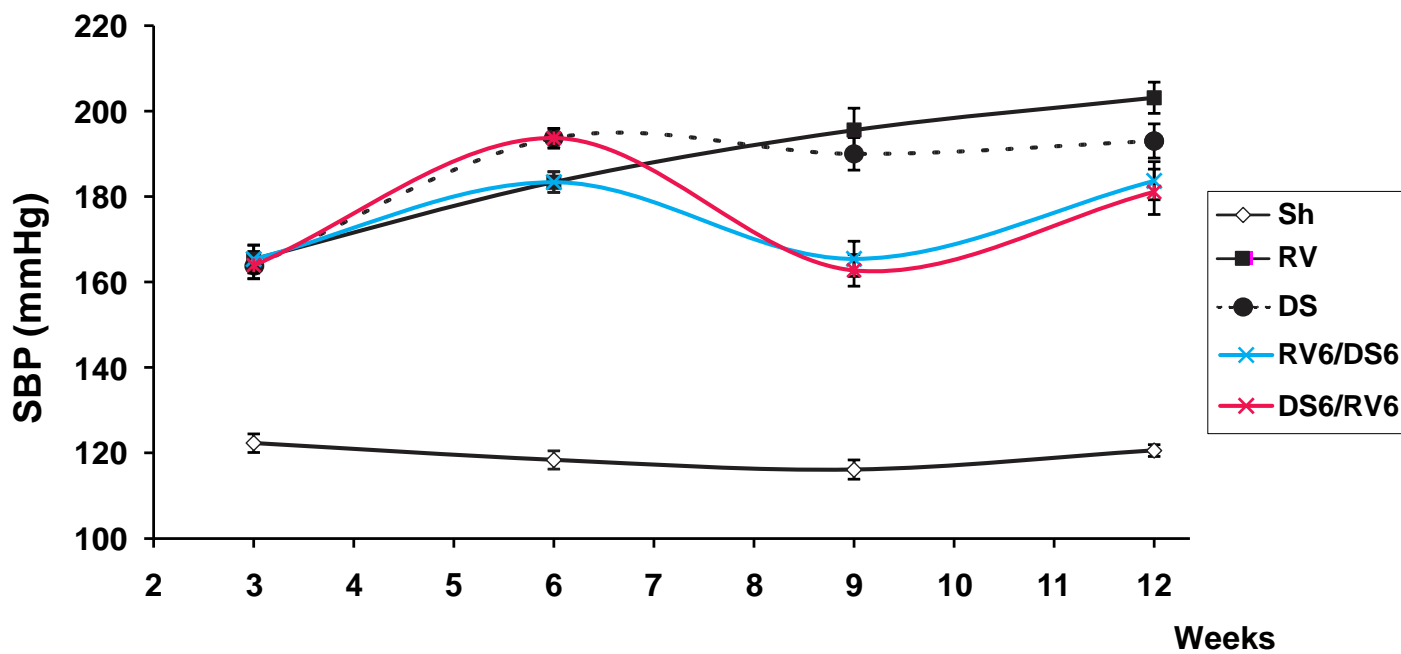

## Supplementary Figure 3

**A**

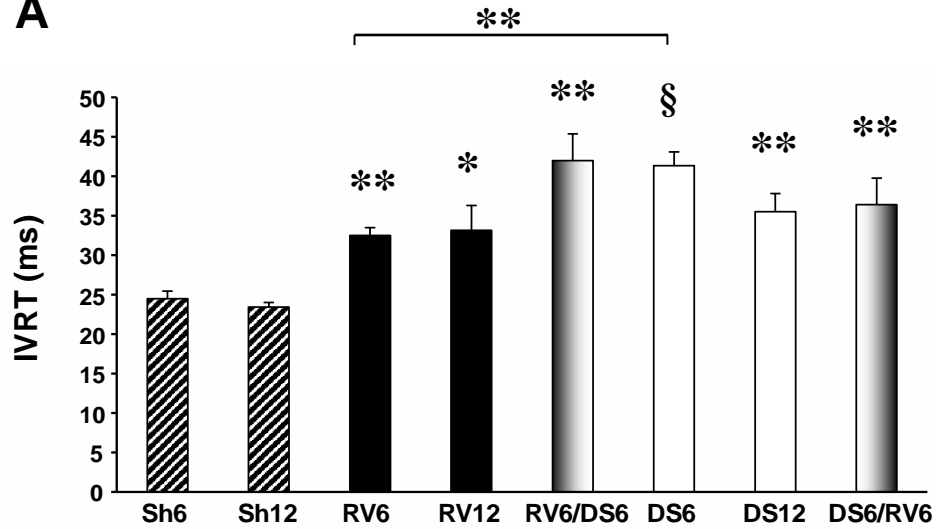

**B**

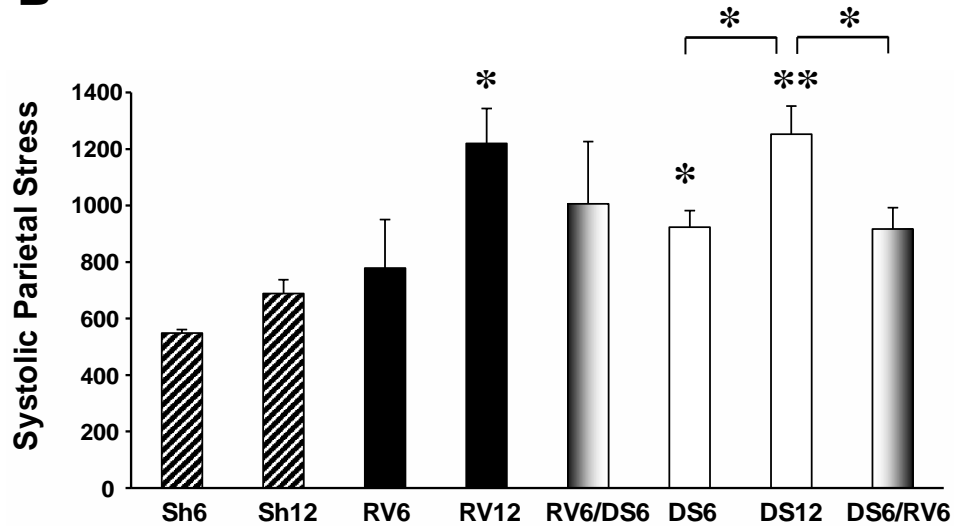

**C**

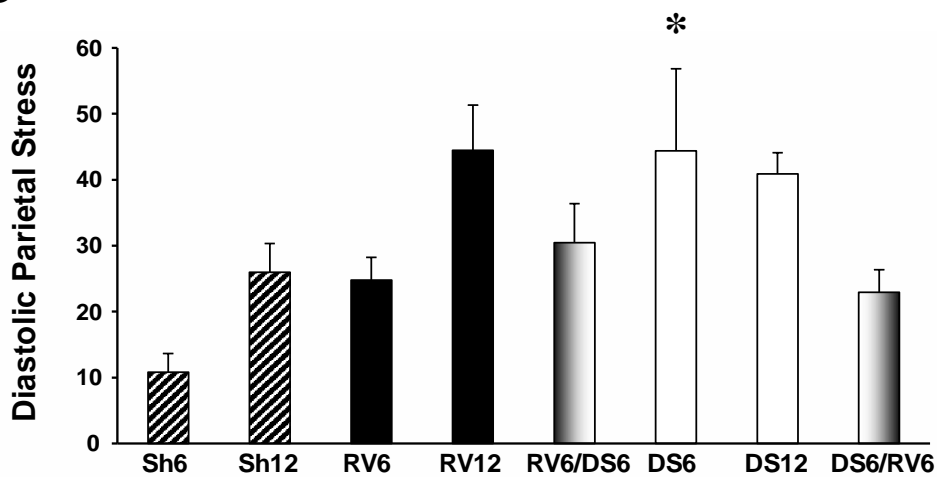

Supplement: Supplementary Figure 1 — Systolic blood pressure (SBP) in RV (upper panel) and DOCA-salt hypertension (lower panel) single treatments compared to their sham at 2, 4, 6, and 12 weeks of treatment. Results are expressed as mean ± SEM. ∗P < 0.01 and ∗∗∗P < 0.001 versus corresponding sham; †P < 0.05 and ‡P < 0.001 versus RV2; §P < 0.01 versus RV6: §P < 0.05 vs. DS2; #P < 0.05 vs. DS4. [file Data_Sheet_1.pdf]
