# Supplementary material for: Cardiac Natriuretic Peptide Profiles in Chronic Hypertension by Single or Sequentially Combined Renovascular and DOCA-Salt Treatments
Source: Front Physiol. 2021 May 25;12:651246. doi: 10.3389/fphys.2021.651246 (PMC8185994; doi:10.3389/fphys.2021.651246)
Supplement: Supplementary Table 1 — Systolic blood pressure (SBP) and hypertrophic indexes in sham animals for different groups. Ratios are calculated in mg tissue/g body weight. Values are expressed as mean ± SEM. BW, body weight; HW, heart weight; LVW, left ventricle weight; RVW, right ventricle weight; LAW, left atria weight; RAW, right atria weight. The number of animals (n) per group is indicated for each parameter. Sh-RV6 and Sh-RV12 are the sham animals for renovascular groups; Sh-DS6 and Sh-DS12 are the sham animals for DOCA-salt groups; Sh-RV6/DS6 and Sh-DS6/RV6 are the sham animals for combined groups. Animals were subjected to simulated surgeries and vehicle injections at the same time points as the experimental groups as indicated in the Methods section. [file Table_1.pdf]

Supplementary Table 1

Group	SBP mmHg	HW/BW mg/g	LVW/BW mg/g	RVW/BW mg/g	LAW/BW mg/g	RAW/BW mg/g
**Sh6**						
**Sh-RV6**	114 ± 3	2.55 ± 0.03	1.90 ± 0.04	0.45 ± 0.01	0.11 ± 0.01	0.10 ± 0.01
**Sh-DS6**	119 ± 2	2.61 ± 0.05	1.97 ± 0.04	0.42 ± 0.02	0.12 ± 0.01	0.11 ± 0.01
**Sh12**						
**Sh-RV12**	120 ± 3	2.56 ± 0.22	1.77 ± 0.02	0.52 ± 0.01	0.12 ± 0.01	0.10 ± 0.01
**Sh-DS12**	120 ± 2	2.38 ± 0.06	1.71 ± 0.02	0.52 ± 0.01	0.11 ± 0.01	0.10 ± 0.01
**Sh-RV6/DS6**	118 ± 2	2.40 ± 0.03	1.65 ± 0.05	0.51 ± 0.04	0.12 ± 0.01	0.10 ± 0.01
**Sh-DS6/RV6**	122 ± 3	2.35 ± 0.13	1.74 ± 0.02	0.55 ± 0.02	0.11 ± 0.01	0.09± 0.01
n	*4-7*	*4-7*	*3-5*	*3-5*	*3-5*	*3-5*
