# Supplementary material for: Cardiac Natriuretic Peptide Profiles in Chronic Hypertension by Single or Sequentially Combined Renovascular and DOCA-Salt Treatments
Source: Front Physiol. 2021 May 25;12:651246. doi: 10.3389/fphys.2021.651246 (PMC8185994; doi:10.3389/fphys.2021.651246)
Supplement: Supplementary Table 2 — Regression of hypertension and cardiac hypertrophy at 12 weeks after withdrawal of DOCA-salt treatment for 6 weeks. A group of animals were subjected to left nephrectomy and DOCA/salt administration as described in the Methods section; after 6 weeks the treatment was discontinued and the parameters were evaluated at 12 weeks. Values are expressed as mean ± SEM. Number of animals (n) is indicated between brackets for each group. §P < 0.001 vs corresponding sham; @P < 0.001 vs DS6; $P < 0.01 and #P < 0.001 vs DS12; ∗P < 0.05 vs DS6/RV6. [file Table_2.pdf]

Supplementary Table 2

Groups	SBP (mmHg)	HW/BW (mg/g)
**Sh6 (11)**	118 ± 2	2.56 ± 0.05
**Sh12 (21)**	121 ± 1	2.46 ± 0.05
**DS6 (16)**	194 ± 2 §	3.89 ± 0.11 §
**DS12 (15)**	193 ± 4 §	4.31 ± 0.16 §
**DS6/RV6 (16)**	181 ± 5 §$	3.37 ± 0.08 §#
**DS6/discontinued (6)**	163 ± 4 §@#*	2.63 ± 0.04 @#*
